# Early nutrition and ageing: can we intervene?

**DOI:** 10.1007/s10522-017-9691-y

**Published:** 2017-03-29

**Authors:** Daniella Duque-Guimarães, Susan Ozanne

**Affiliations:** 1 MRC Metabolic Diseases Unit, Addenbrooke’s Hospital, Wellcome Trust-MRC Institute of Metabolic Science, University of Cambridge Metabolic Research Laboratories, Cambridge, CB2 0QQ UK; 2 Department of Physiology and Biophysics, Institute of Biomedical Sciences, University of São Paulo, São Paulo, 05508-000 Brazil

**Keywords:** Ageing, Fetal programming, Early nutrition, Lifespan, Intervention

## Abstract

Ageing, a complex process that results in progressive decline in intrinsic physiological function leading to an increase in mortality rate, has been shown to be affected by early life nutrition. Accumulating data from animal and epidemiological studies indicate that exposure to a suboptimal nutritional environment during fetal life can have long-term effects on adult health. In this paper, we discuss the impact of early life nutrition on the development of age-associated diseases and life span. Special emphasis is given to studies that have investigated the molecular mechanisms underlying these effects. These include permanent structural and cellular changes including epigenetics modifications, oxidative stress, DNA damage and telomere shortening. Potential strategies targeting these mechanisms, in order to prevent or alleviate the detrimental effects of suboptimal early nutrition on lifespan and age-related diseases, are also discussed. Although recent reports have already identified effective therapeutic interventions, such as antioxidant supplementation, further understanding of the extent and nature of how early nutrition influences the ageing process will enable the development of novel and more effective approaches to improve health and extend human lifespan in the future.

## Ageing

According to the last WHO report (WHO 2015), the proportion of elderly people is increasing globally. The increase is not only observed in developed countries but also in countries still undergoing development such as China, India and Brazil. Projections for 2050 indicate that 30% or more of the population in many places in Europe, America and Asia will be over 60 years old. From a biological point of view, ageing is a complex process associated with the accumulation of damage at molecular, cellular and tissue levels; therefore the drastic increase in the number of elderly people will also bring an increased risk of many diseases in the population. Most people over 65 years show an exponential increase in the risk of developing two or more age-associated diseases such as cancer, cardiovascular disease, neuro-degeneration and type 2 diabetes. This not only has an impact on the individual and their family, but also health care systems (WHO 2015).

It is important to highlight that ageing happens based on a combination of factors, and the consequences of its progression varies between individuals. The considerable increase in human life expectancy in recent history makes it clear that, although genetic factors are thought to be responsible for about one-third of the variation in life expectancy, the influence of the environment and behaviour strongly affects the development of age-associated disease and our lifespan.

One of the biggest challenges nowadays is to ensure that, with the increase in lifespan, there is also a parallel increase in health-span. It is therefore important to find a way to delay ageing progression and thus, reduce the prevalence of associated diseases. It is thus becoming crucial for researchers to focus on factors that could make a difference to how healthily we age. Diet, genes and drugs are generally the most studied topics in the literature addressing factors that influence health and longevity. There has been great interest in elucidating molecular mechanisms underlying the effects of diet on lifespan, as these could give insight into possibilities for new treatments. Dietary regimens enforcing caloric restriction, in which calorie intake is reduced but not to the extent of malnutrition, are one of the oldest and most reproducible means of increasing lifespan and reducing age-associated disease risk (MacDonald and Ramsey 2010). The association between caloric restriction and lifespan has been demonstrated in a variety of different organisms including rodents, worms, yeast and flies (Barrows and Kokkonen 1982; Weindruch et al. 1986; Most et al. 2016). One of the first studies showing clear evidence that caloric restriction could increase longevity and protect against the development of age-associated diseases dates back to the 1930s (McCay et al. 1935). Since then, interest in this area has grown with the results of many studies in animals demonstrating the benefits of a reduction in caloric intake to extend lifespan, with preservation of a “youthful” phenotype due to the reduced incidence of age-associated diseases. For instance, in 2009, an elegant study of nonhuman primates showed adult-onset moderate caloric restriction could delay the development of age-related diseases and extend lifespan (Colman et al. 2009). These results were later contradicted by another study in nonhuman primates (Mattison et al. 2012), demonstrating that the effects of caloric restriction on health and ageing are confounded by other environmental and genetic factors. However, although complex owing to the differences in study design, more recently when the findings of these two studies were analysed together it was concluded that caloric restriction initiated in adulthood is indeed beneficial for health and possibly longevity (Mattison et al. 2017).

It is not clear if caloric restriction also extends life span in human subjects. However, it has been documented that dietary restriction prevents type 2 diabetes and hypertension, and reduces risk factors for the development of cancer and cardiovascular disease (Heilbronn and Ravussin 2003; Longo and Fontana 2010). In general, nutrition appears to exert a substantial influence on lifespan and the development of age-associated diseases. Although current diet is known to be an important determinant of health, recent studies have suggested that diet during critical periods of development, such as fetal and early neonatal life, may also be important in determination of health span and lifespan (Tarry-Adkins and Ozanne 2014).

## Fetal origins hypothesis

Professor David Barker, a British physician and epidemiologist, proposed almost three decades ago the ‘Fetal Origins’ hypothesis, now commonly referred to as the developmental origins of health and disease hypothesis. The hypothesis suggested that exposure to a suboptimal nutritional environment during fetal life can have long term effects on adult health, contributing to the development of age-associated diseases (Barker 1992). Some of the earliest evidence in support of this hypothesis came from the study of men living in Hertfordshire, UK, for whom birth weights and current health data were available. This demonstrated that men with the lowest weights at birth and at 1 year of age had the highest death rates from coronary heart disease (Barker et al. 1989). As part of the fetal origins hypothesis, Barker, along with his colleague Nick Hales, went on to demonstrate that there are also associations between poor fetal growth and the subsequent development of type 2 diabetes and metabolic syndrome in adulthood (Hales et al. 1991; Barker et al. 1993). The researchers proposed that these relationships arose because of the response of the growing fetus to exposure to under-nutrition in utero. This response included prioritising development of vital organs such as the brain, at the expense of the growth of other organs such as the endocrine pancreas. Changes to organ development and programmed changes in cellular metabolism (in a manner geared towards efficient nutrient storage) would have long term consequences in how the organism is able to store and utilise nutrients. These changes were proposed to be beneficial for short- term survival if the fetus was born into conditions of continued under-nutrition, but become detrimental in conditions of adequate- or over- nutrition post-natally. Many studies were published confirming this concept that became known as the thrifty phenotype hypothesis (Phillips 1994; Mericq et al. 2005; Hales and Barker 1992; Norris et al. 2012; Duque-Guimarães and Ozanne 2013).

The Dutch Hunger Winter study is one of the most important examples from a human context that supports the idea of programming by early nutrition. During the Second World War there was a period of time when food supplies were cut off to the western part of the Netherlands, resulting in rationing of food to as little as 400–800 calories/day to the population, including pregnant women. The nutritional limitation imposed on the women during pregnancy, followed by a rapid increase in prosperity in the post-war period, had long-lasting consequences for the adult health of the offspring who were in utero during the famine, including increased risk of glucose intolerance and cardiovascular disease (Ravelli et al. 1998; Roseboom et al. 2000, 2001). Further evidence in support of the fetal origins hypothesis was obtained from a study of monozygotic (identical) twins showing that when there was discordance for type 2 diabetes in adulthood among twins, the diabetic twin had a lower birth weight than their non-diabetic co-twin (Poulsen et al. 1997).

Experimental studies have also provided strong evidence that early-life nutrition is an important factor in determining the long-term health of an individual. This type of study is important, as it also provides a better understanding of the underlying mechanisms. For instance, we showed some years ago, using adipose tissue and skeletal muscle biopsies from humans of known birth weight, that low birth weight is associated with reduced levels of insulin signaling proteins in both tissues (Ozanne et al. 2005, 2006).

Although the first studies in relation to the fetal origins of disease mainly focused on the consequences of nutritional deprivation during pregnancy, the impact of a hyper-caloric diet and increased maternal body weight during pregnancy has recently gained a lot of attention due to the rapid increase in obesity among women of child-bearing age. It is now known that the consequences of maternal obesity during pregnancy are very similar to those observed as a consequence of maternal under-nutrition during pregnancy. These include increased risk of developing age-associated diseases such as obesity, cardiovascular disease and type 2 diabetes in adulthood (Li et al. 2011; Poston 2012; Alfaradhi et al. 2014; Blackmore et al. 2014; Alfaradhi et al. 2016).

## Can early nutrition impact on lifespan?

Many research groups have used experimental models to investigate the effects of various sub- optimal nutrition states during early life on later life disease risk. These include maternal protein restriction (Langley-Evans 1999; Petry et al. 2001), maternal iron restriction (Lewis et al. 2001), maternal uterine ligation (Simmons et al. 2001), maternal caloric restriction and maternal obesity (Samuellson et al. 2008). These differing models have all shown effects in the offspring in terms of development of metabolic disturbances and age-associated diseases such as type 2 diabetes and hypertension. There are therefore clear effects of early nutrition on health span. However, the data on potential effects on lifespan is much more limited. We have been one of the few research groups to address this issue. Using a mouse model, we investigated the effects of maternal protein restriction during either pregnancy or lactation on lifespan, and also addressed how effects were modulated by a post-weaning obesogenic diet. We demonstrated that exposure to a low protein diet during fetal life reduced life span, and that longevity was further reduced when animals were weaned onto an obesogenic diet. In contrast, maternal protein restriction during lactation slowed the growth of neonates and increased their lifespan. In addition these offspring were resistant to diet-induced obesity and therefore were protected from the detrimental effects of an obesogenic diet on lifespan (Ozanne and Hales 2004, 2005). These data demonstrate that the timing of the nutritional interference is crucial, and highlight the possibility that an understanding of the mechanisms underlying these programming effects on life span will help develop interventions during critical periods of development that could reduce the incidence of age-associated diseases and improve longevity. Although human data on this matter is rare, Abeelen and colleagues have reported, based on data from the Dutch Famine Birth cohort, that under-nutrition during fetal life in humans may also impact on lifespan (Van Abeelen et al. 2012).

The precise molecular mechanisms underlying the long-term effects of nutritional changes during early life on the longevity and development of age-related diseases are still not clear. However, animal studies have provided valuable insights into how an unfavorable prenatal environment triggers programming effects in the offspring. These include permanent structural changes to organs, alterations in gene expression (possibly via alterations in epigenetic modifications) and oxidative stress that can lead to an accelerated cellular ageing.

There is much evidence demonstrating that early nutrition can lead to permanent changes in the structure of tissues, resulting in an ageing phenotype and development of age-associated diseases. For instance, it is widely recognized that suboptimal early nutrition leads to increased risk of type 2 diabetes (Duque-Guimarães and Ozanne 2013), and one possible mechanism underlying this could be through effects on the structure and consequently function of the endocrine pancreas. In a model of maternal protein restriction during gestation, it has been shown that the offspring had smaller pancreatic islets, impaired B cell proliferation and insulin release as well as a reduction in islet vascularization and increased age-associated development of islet fibrosis (Snoeck et al. 1990; Tarry-Adkins et al. 2010). There is also literature demonstrating that suboptimal nutrition in early life can lead to lower nephron number and size and cardiac remodeling, both of which would influence cardiovascular health (Merlet-Benichou et al. 1994; Woods et al. 2001; Blackmore et al. 2014).

Epigenetic mechanisms have received a lot of attention in the developmental programming field as a likely mediator of permanent changes in gene expression. DNA methylation, histone modification and non-coding RNAs are the main described epigenetic mechanisms, and cause changes in gene expression without changing the DNA sequence (Bird 2007; Margueron and Reinberg 2010). It has been demonstrated that in utero exposure to a low protein diet can lead to changes in DNA methylation and histone modifications that affects the expression of important transcription factors such as HNF4α, PPARα and CEBPb (Sandovici et al. 2011; Slater-Jefferies et al. 2011; Zheng et al. 2011). More recently, Dobson and colleagues showed that high sugar diets in early life program fly and worm lifespan through regulation of the FOXO transcriptional factor (Dobson et al. 2017). Similarly, maternal diet-induced obesity leads to changes in mRNA translation through alterations in microRNA expression (Fernandez-Twinn et al. 2014). In addition, Heo and colleagues demonstrated that early developmental exposure to either maternal under-nutrition or a maternal western diet leads to transcriptional dysregulation of important metabolic pathways and alters the methylation profile in offspring in a manner very similar to that usually associated with ageing (Heo et al. 2016).

There is evidence to suggest that some of the mechanisms that are generally associated with cellular ageing/senescence may be involved in mediating the detrimental effects of suboptimal early nutrition on longevity. One potential process is through alterations in telomere length. Telomeres are hexameric repeat sequences at the ends of chromosomes that protect genetic material from degradation and are considered to be important markers of senescence. They shorten as a consequence of cell division in most somatic cells, but also as a consequence of oxidative stress. Cellular ageing is associated with telomere shortening, which may cause irreversible replicative senescence and therefore apoptosis (Harley et al. 1990; Bernadotte et al. 2016; Fairlie et al. 2016).

Experiments in our laboratory have shown that offspring from a maternal protein restriction model, in which rats are exposed to a low-protein diet in utero and then suckled by normally fed dams to induce catch-up growth, demonstrate reduced life span. These animals also display an accelerated cellular ageing phenotype with increased levels of oxidative stress, senescence markers and accelerated telomere shortening in a range of tissues including pancreatic islets and heart (Tarry-Adkins et al. 2009, 2013). There are no current reports from animal models of the potential effects of maternal obesity on offspring telomere length. However, there is evidence that maternal obesity is associated with mitochondrial dysfunction and increased oxidative stress in the offspring, which are consistent with an accelerated ageing phenotype (Alfaradhi et al. 2014; Bayol et al. 2010). Recently, Martens and colleagues have reported in humans a strong negative association between pre-maternal BMI and telomere length in newborns (Martens et al. 2016). Although it is not clear if this association is causative, it consistent with a potential role of accelerated cellular ageing in individuals exposed to sub-optimal early life nutrition.

Many of the above models have discussed the effects of maternal nutrition during pregnancy on age- related disease outcomes in the offspring, However, there is also emerging evidence that paternal nutritional state at the time of conception can program offspring health (Ng et al. 2010, 2014; McPherson et al. 2015). These effects are thought to be mediated by transmission of genetic material such as small non-coding RNAs in sperm (Fullston et al. 2016; Grandjean et al. 2015). There is evidence that these paternal effects can be reversed by exercise and/or weight loss in both humans and animal models (Donkin et al. 2016; McPherson et al. 2015).

## Intervention strategies

The concept that nutrition during critical early periods of development programs offspring predisposition to a wide variety of age-associated diseases is now generally accepted. However, a better understanding of the best intervention strategies to revert the detrimental consequences of nutritional programming on offspring health requires further attention. This will be greatly aided by studies of experimental interventions in animal models in the setting of early nutrition and longevity, in which the intervention and other environmental and genetic variables can be tightly controlled.

It has been demonstrated that ageing is associated with complex epigenetic changes at the transcriptional and translational levels. An increase in cellular oxidative stress may be one of the main factors that induces these changes, since disturbances in the normal redox state of cells is a major phenotype of the ageing process and some of the main epigenetic modifying enzymes are redox-sensitive (Benayoun et al. 2015). An imbalance in the generation of reactive oxygen species and the antioxidant capacity of the organism is also a known consequence of a suboptimal early nutritional environment (Thompson and Al-Hasan 2012). Therefore, reducing cellular oxidative stress is one approach that has been adopted in an attempt to prevent the detrimental effects of developmental programming. Several groups have shown potential reversibility or delay in the programmed accelerated ageing process using nutritional supplementation (Sen and Simmons 2010) or other types of interventions in the pregnant mother such as exercise (Vega et al. 2015) and pharmacological approaches (Cambonie et al. 2007).

The animal studies described above focus on interventions during pregnancy, which would target fetal development in utero. However, in terms of translatable intervention studies that can be used in humans, intervention to the offspring themselves after birth may be more useful as this can be targeted to individuals who have experienced sub- optimal early life nutrition. This is particularly important as some markers of in utero fetal nutrition, such as birth weight, are by definition not apparent until after birth. Data from our laboratory, using the maternal low protein rat model, has demonstrated that post weaning supplementation of the offspring diet with coenzyme Q10, (an important endogenous antioxidant and a key component of the electron transport chain) at least in part reverses many of the consequences of nutritional programming, including effects on cardiac, hepatocyte and adipocyte ageing, inflammation, telomere shortening, DNA damage, cellular senescence and apoptosis, and insulin resistance (Tarry-Adkins et al. 2013, 2014, 2015, and 2016).

## Conclusion

It is widely recognized that early life nutrition can exert long-term effects in adulthood, including the risk of developing many age-associated diseases as well as impacting on lifespan. This idea is overwhelmingly supported by a large amount of evidence from different nutritional conditions, including under- and over- nutrition during fetal life, in both animal models and human cohorts. Whilst nutritional programming is a multi-factorial process and occurs as a consequence of both under- and over- nutrition, the variety of models with a common end-point might suggest some common mechanisms. Certainly there appears to be a role for an accelerated cellular ageing process, involving oxidative stress and permanent structural alterations associated with epigenetic changes. Potential strategies targeting these mechanisms, in order to prevent or alleviate the harmful effects of suboptimal early nutrition on lifespan and age-related diseases, include antioxidant supplementation and exercise. However, further understanding of the extent and nature of how early nutrition influences the ageing process could enable the development of novel and more effective approaches to intervene in the future.
